# Psychosocial indicators of individual behavior during COVID 19: Delphi approach

**DOI:** 10.1057/s41599-022-01363-6

**Published:** 2022-09-30

**Authors:** Wijdan Abbas, Shahla Eltayeb

**Affiliations:** Naif Arab University for Security Sciences, Riyadh, Saudi Arabia

**Keywords:** Sociology, Psychology, Health humanities

## Abstract

The COVID-19 pandemic revealed the weakness of the health care system to incorporate indicators of human behavior in the rapid response to the virus. This study aims to establish consensus on the psychosocial indicators of COVID-19 preventive behaviors during the initial phase of the outbreak in Arab countries. This qualitative study used a combined scoping review of the literature to develop the 24 psychosocial indicators and the Delphi approach with a panel of 27 experts from nine Arab countries to achieve a consensus on preventive behavior indicators. The most robust agreement with an average rating of at least 4 was found for five social indicators including Belief System with an average rating (5). Income Status average rating (4.9). Family Commitment average rating (4). Faith average rating (4). Kinship System average rating (3.9). Four psychological indicators were identified namely Self-Efficacy with an average rating of (5). Perception of Hazard with an average rating of (4.5). Motivation with an average rating of (4.5). Stigma with an average rating of (4.2). The nine indicators provide a strong base for redesigning pandemic control interventions. The Delphi study demonstrates the feasibility of a participatory approach during the outbreak of COVID-19. Moreover, future interventions need to accommodate individual psychological and social determinants to increase adherence and decrease resistance to public health guidelines.

## Introduction

Arab countries responded to the COVID-19 pandemic by allocating resources, implementing policies, imposing strict procedures, measures, and imposing penalties. Still, the result of controlling the virus and preventing its spread was not consistent with the efforts made to confront the crisis. A study by Kandel et al. (2020) indicated that out of 182 countries, 28% have a fragile level of preventive capabilities, and 33% of countries have weak response levels; this finding demonstrates that countries were operationally ready but did not have effective predictive capabilities to curb public health risks (Emanuel et al., 2020; Wright et al., 2020; Blustein et al., 2020). The weakness in considering individuals’ social and psychological characteristics made the prevention strategies for the COVID-19 virus deficient, as prevention strategies were based initially on one policy for all without considering social and psychological differences. Wang and Wang (2020), study dealt with weaknesses, strengths, threats, and opportunities in dealing with the COVID-19 pandemic in China; they pointed out that there was a lack of interest in social pressure factors and a lack of adherence to preventive measures. In addition to ignoring rumors and their spread and increasing fear and anxiety associated with the ban. These factors have weakened the public health model to deal with the pandemic.

Exploring individuals’ risk perceptions is critical to understanding their response, behavior, and adoption of preventive measures at the individual level (e.g., wearing masks, and washing hands) in the event of an infectious disease outbreak and its consequences. Identifying risk perceptions will not only help mitigate the devastating burden of mortality and disease, but also economic loss. Several studies focused on the effects of the COVID-19 pandemic, whether on individuals (Cao et al., 2020) or the economic impact resulting from the pandemic (Breisinger et al., 2020). Other studies were concerned with the psychological impact of the pandemic and traced the social and health effects of the pandemic as a study Bodrud-Doza et al. (2020) and Tull et al. (2020). Al-Zahrawi (2020) analyzed the security and political impact of the pandemic. However, no single study combined social and psychological indicators to predict preventive behavior towards the pandemic. The current prospective study aims to answer the main question: What psychosocial indicators among Arab countries contribute to individual adherence to preventive behavior during COVID-19?

The importance of this study comes from its significance on the theoretical and applied levels in epidemics prevention in general and the emerging COVID-19 in particular. The study would enable decision-makers in Arab countries to identify the psychosocial indicators of individuals adhering to health guidelines during pandemics.

## Theoretical framework

Models and theories related to public health represent the basis for psychological and social ideas that explain the preventive behavior of the emerging COVID-19. Reviewing behavioral change principles that vary across several explanatory models and theoretical rules indicated more than 22 theories and 60 models (Gehlert and Ward, 2011). That analyzes the factors and reasons for constructing behavioral preferences according to knowledge, values, beliefs, intentions, motivation, etcetera. This study operationalizes Socio-psychological theories concerned with explaining behavioral change integrative behavior theories; it combines the internal and external factors of the individual and society, allowing for a more comprehensive analytical understanding of preventive behavior among middle eastern communities. The following section will review models associated with behavior change, including the Health Belief Model, Social Engineering Theory, and Theory of Planned Behavior Control.

The Health Belief Model (Janz and Becker, 1984) emphasizes the role of people’s awareness, perceived risk, self-efficacy, and feasibility of preventive measures in explaining individual health behaviors. Undoubtedly, adopting social distancing and adhering to preventive behaviors as strategies in confronting COVID-19 requires individual and collective behavior committed to this strategy and keen on it in all cases. Without this commitment, these strategies will not succeed. The evidence indicated that many weaknesses and disparities marred the response to social distancing and adherence to preventive measures at the level of countries and cities, and regions in a single country (Rohwerder, 2020). Studies from the Arab region have shown that despite international and national health guidelines, coronavirus prevention behaviors in the general population are still unsatisfactory (Karimy et al., 2021). The Health Belief Model assumes that individuals will adhere to preventive health behaviors if they perceive the pandemic as threatening (perceived susceptibility) or COVID-19 as severe with serious health complications (perceived severity). With more information and awareness, people may consider adopting preventive behaviors (calls to action), they trust the usefulness of preventive behaviors (perceived benefits), and the benefits outweigh the perceived costs and barriers (Walrave et al., 2020).

While Social Engineering Theory is concerned with changing harmful health behaviors through social engineering, which focuses on modifying the individual’s environment, thus influencing their ability to practice healthy, positive, and appropriate behavior. This theory relies on the influence of the surrounding environment more than direct programs to change behavior. The theory does not require only abstaining the individual from a specific behavior, which is considered negative action; instead, it involves the practice of positive, healthy behavior to modify negative behavior. For example, most Arab countries in the Gulf area use temperature measurements at the entrance to shops and workplaces, and a ban imposed on gathering places are positive measures to prevent COVID-19 exposure. In this way, the behavior promoted had been determined to occur due to social engineering, Thus, sometimes, the solutions provided by social engineering to health problems are more successful than individual solutions (Suyanto et al., 2020).

According to the Theory of Planned Behavior, individual behavior is guided by three types of considerations: Behavioral beliefs, i.e., the personal probability that this behavior will lead to an inevitable outcome. Normative beliefs are the perceived behavioral expectations of individuals or important reference groups in the individual’s environment and the motivation to meet those expectations. Control beliefs are associated with the existence of factors that can support or hinder performing the behavior and the perceived strength of these factors in direct proportion to the perceived behavioral control with the individual’s personal probability that the control factor exists. The behavioral intention had formed through these three considerations (Largo-Wight et al., 2012). A study by Frounfelker et al. (2021) indicated that TPB constructs were positively associated with the intention to follow social distancing guidelines. While perceived social isolation and fear of COVID-19, discrimination was associated with poor adherence. Hence during the COVID-19 pandemic, individuals’ sense of compliance with health guidelines would be affected by their beliefs, attitudes, and perception of one capability that they can comply.

The current study explored the above theories to identify social and psychological indicators in the prevention of COVID-19 among the Arab population. One can postulate that COVD 19 prevention efforts focused on promoting individual control beliefs by focusing on personal responsibility in hand wash and wearing a facemask. While other efforts focused on social engineering as curfew and travel bans, others used fear appraisal by emphasizing the fatality of COVID-19. Nevertheless, most of the prevention efforts methods did not holistic approach to multifactor psychosocial factors that could assist in a predictive framework of human behaviors during pandemics. The balance between promoting healthy behavior, personality, and collective behavior and the social environment requires an integrated awareness of psychological and social indicators.

### Contextual understanding of COVID-19 Preventative Behavior in Arab Countries

Modifying individual behaviors during a pandemic can reduce the impact of the outbreak rather than eliminating the disease itself. However, in order to develop health interventions to mitigate pandemics, it is important to understand individual behaviors. The adoption of preventive measures is also deeply rooted in the social-ecological context. Studies in the U.S. and the U.K. have indicated that migrants and ethnic minorities relatively often have difficulty taking the necessary preventive measures because of their employment in important occupations or their relatively large households (Torensma et al., 2021).

Sociocultural and socioeconomic factors in Arab countries can have serious implications for the acceptance of COVID-19 preventive measures. Arab countries share several commonalities in their social norms and practices that reflect their historical, religious, and sociocultural characteristics. They are all predominantly Muslim and speak the Arabic language.

Typically, societies in the Arab cluster have a medium Human Development Index, with oil-producing countries in the Gulf having high incomes. These differences may have influenced individual decisions to adhere to protective COVID-19. A recent study by Bonyan et al. (2020) examined public awareness of COVID-19 in some Gulf Arab countries (Saudi Arabia, Emirates, and Qatar), where excellent economic conditions enabled countries to adopt protective measures, including school and workplace closures, strict travel bans, and strong business economic support. In contrast, middle- to low-income Arab countries have struggled to enforce COVID-19 health guidelines due to the population’s reliance on daily labor income, limited social support systems, and inadequate Internet services, which can hinder awareness efforts. In Egypt, Abdelhafiz et al. (2020) recognized a positive general attitude toward measures that can be followed to prevent the transmission of COVID-19. However, less than 30% are willing to put on a facemask to protect themselves from infection or comply with collection restrictions. Another study found that although the government mandates preventive measures, some of these measures are not followed. This suggests that barriers to compliance with COVID-19 preventive measures need to be urgently addressed and removed in future interventions to prevent the spread of COVID-19 among Egyptians.

Differences in protective behaviors from COVID-19 were further identified across Arab countries, Faour-Klingbeil et al. (2021) study further identified culture-specific hand-washing deficits and unsafe food handling practices during COVID-19 with more Tunisians using cleaning agent’s sanitization compared to Jordanian and Lebanese. The current study aims to understand the indicators associated with the adoption of preventive measures against COVID-19 by Arab communities, This requires exploring multiple and complex indicators to unfold the structural social, and psychological barriers that curtail the opportunity to act upon preventive measures.

## Methodology

### Study design

**Fig. 1:**
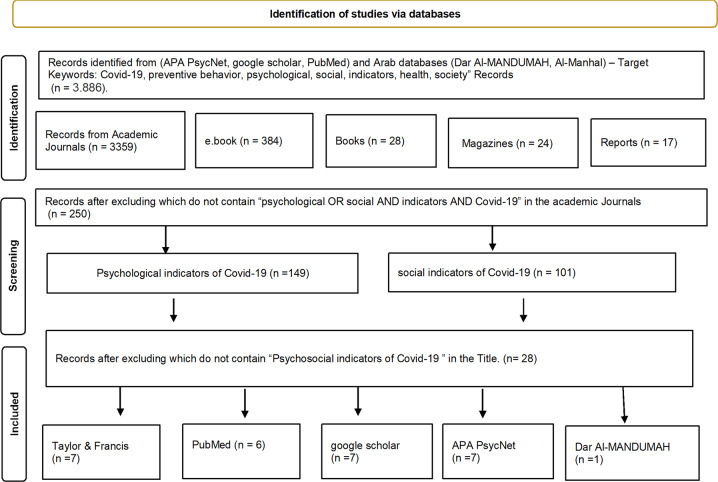
PRISMA Flow Diagram. PRISMA 2020 flow diagram for included searches of databases and other sources.

To determine the psychosocial indicators of COVID-19 preventive behaviors, this study involved two steps: scoping review and a multi-round Delphi survey of experts. A non-systematic scoping review of published scientific articles was used rather than a question-driven systematic review methodology. As the current study focuses on identifying psychosocial indicators. The Scoping Review assisted in identifying potential indicators for preventive health behavior of COVID-19 in Arab and foreign databases such as (psychnet, google scholar, PubMed, Dar Al-Manthama, Al-Manhal). The following keywords were used (Covid-19, preventive behavior, psychological, social, indicators, health, society) individually and collectively (see Fig. 1).

The Delphi technique is a standard method for developing health and education frameworks (Heiko, 2012). In addition to being cost-effective, the Delphi technique also offers advantages in presenting generalized indicators and targets. In this study, a literature review was conducted to develop a foundation of psychosocial indicators of preventive behaviors, and a Delphi consensus method was used. The results of this study were reported according to the guidelines of the Standard for Conducting and Reporting Delphi Studies (CREDES) (Jünger et al., 2017).

### The Consensus during Delphi rounds

**Fig. 2:**
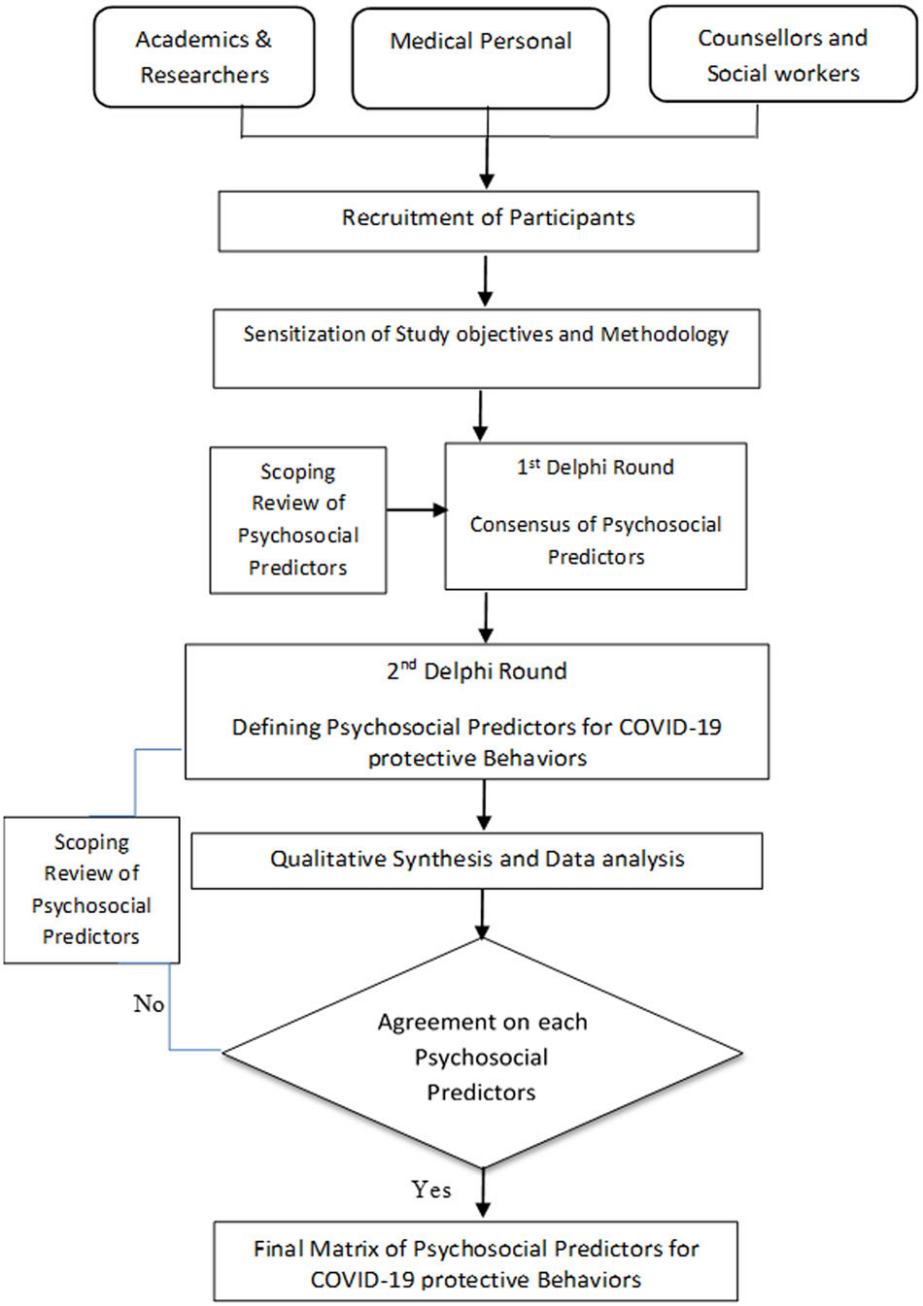
Methodology in the Delphi consensus study, outlines how the Delphi method proceeded through three rounds of consultation with expert panel members in order to achieve consensus. The Delphi process comprised three rounds. In round one, the panelists were presented with 24-items questionnaire using five-point Likert scale The consensus was reached when the absolute deviation was less than one point on the five-point scale (i.e., ±10%). Any indicators for which consensus was not reached at the end of Round 2 were eliminated in Round 3. The survey was continued for three rounds.

The Delphi process comprised three rounds. In round one, the panelists were presented with 24-items questionnaire about the Covid-19 psychosocial indicators employing the Likert scale of 1–5, described as follows: 1—Little important, 2—Somewhat important, 3—Important, 4—Very important, 5—Most important. These indicators were divided into two main categories, with each category containing sub-indicators (12 social indicators and 12 psychological indicators). The consensus was reached when the absolute deviation was less than one point on the five-point scale (i.e., ±10%). The consensus was measured by the average absolute deviation from the median, which is the absolute deviation that fell below one point on the five-point scale, (see Table 2). The average and median ratings indicated the strength of the indicator. In the second round, we compiled a summary of the results from the panelist consensus. The goal was to allow panelists to consider the reasons for different responses in order to reduce variability in responses and achieve group consensus. Any indicators for which consensus was not reached at the end of Round 2 were eliminated in Round 3. The survey was continued for three rounds (see Fig. 2), as previous research has shown that this is the optimal round for Delphi studies (Rajhans et al., 2020)

### Delphi panel recruitment

In general, there is agreement on the required number of experts for a Delphi study (Liamputtong, 2019), Considering the participatory nature of this study, using a non-probability sampling we targeted experts in human behavior (with more than 20 years of experience) from 9 Arab countries (Saudi Arabia, Egypt, Tunisia, Algeria, Kuwait, Jordon, Yamen, Syria, and Sudan). Out of the 27 invited experts, 19 panelists completed the three rounds of the Delphi process. This number is in line with recommended Delphi techniques (Heiko, 2012). Arab experts and scholars were contacted by telephone, they were introduced to the study objectives and method, and were assured of anonymity. It was emphasized that the information provided by the panelists would be kept confidential, and used for research purposes only, and informed consent was obtained, completing the recruitment process. Analysis of qualitative data was conducted at the end of each round to avoid the risk of bias from individual contributions and opinions.

### The Delphi procedure

The Delphi process was conducted online, so there was no need to meet in-person or travel to the study. All communication took place via email. Panelist input was stored on online data repositories.

## Results

The scoping review identified 101 studies that examined social indicators of Covid-19, While 149 studies explored the psychological indicators of Covid-19. Studies that examined the combined Psychosocial indicators of Covid-19 were (28) studies. See details in Fig. 1. The current study extracted from previous studies the social and psychological indicators of preventive behavior to formulate the 24-items questionnaire.

The modified Delphi approach suggested by Rajhans et al. (2020) uses a structured questionnaire with a Likert scale instead of open-ended questions, this also facilitates the coding of terminologies and naming of the different indicators. The first round of consensus in the Delphi process ensured the applicability of these indicators in the context of COVAID 19 preventive behaviors.

The panelist represented nine Arab countries and different experts related to social sciences. Table 1 shows the current professional roles and diversity of experience of the Delphi panelists. The Delphi process to define and validate the psychosocial indicators was conducted in three rounds.

**Table 1:** The job role and number of panelists.

Present job role	Number of panelists		Scholar’s title
Academics	Psychology and Sociology	3	Professor
		2	Associate Professor
Academics	Media	4	Assistant Professor
Clinician	Mental health	5	MD, Psychiatrist
Practitioner	Social worker	2	Professor
		3	Senior SW at the ministry of health
Countries included	Syria	2	
	Saudi Arabia	3	
	Egypt	3	
	Sudan	2	
	Tunisia	2	
	Algeria	2	
	Yamen	1	
	Jordan	2	
	Kuwait	2	

The consensus was reached after the third round for 9 of the 24 indicators assessed (see Tables 1 and 2), had a minimum rating between 5 to 3.9, the indicators included five social indicators and four psychological indicators. The average deviation and range of responses show the extent to which the panel agreed with the median value. The lower consensus among panel members is measured by higher deviation, the range of responses was also important in determining how varied the responses were. Although the panelists agreed that the indicators identified in the scoping analysis were significant, there was consensus on less than half of the indicators. In some cases, the lack of consensus was due to minor differences in ratings; in other cases, there were wide differences in views. The indicators where panelists did not reach a consensus are also important for further research and analysis.

**Table 2:** The social indicators of individual COVID 19 preventive behavior.

Social indicators	Items	Average rating	Median rating	Average deviation	Minimum	Maximum
	Family commitment: the commitment of all family members to follow all health preventive guidelines	4.0	4	0.36	3	5
	Family social responsibility: elevated Family sense of responsibility toward others	3.5	3	0.64	1	5
	Kinship ties: participating in social events and kinship ties is as important as health preventive guidelines	3.9	4	0.27	3	5
	Belief system1: destiny is what God will happen despite preventive measures	5.0	5	0.0	3	5
	Faith: infections and disease are tests from God to individual faith	4.0	4	0.08	2	4
	Religiosity: corona prevention falls under the Islamic principle (do not throw oneself to destruction)	2.0	2	0.55	1	3
	Conspiracy: coronavirus is an unreal and propaganda governments	3.5	3	0.45	3	5
	Trust: preventive health guidelines are trustworthy	2.0	2	0.55	1	3
	Political: rejecting the preventative health guidelines is a form of political resistance	3.5	3	0.09	2	5
	Income: fear of losing daily livelihood income if you follow the preventive measures	3.0	3	0.27	3	5
	Cost: buying (sterilizers and masks) is expensive	2.0	2	0.55	1	3
	Job security: preventive measures will lead to job losses	4.9	5	0.17	3	5

### Social indicators

Five social indicators within the social domain had been rated by the panelist as predictors for CoviD-19 behaviors (see Table 2), these include the Belief System with an average rating of (5). Income Status average rating of (4.9). Family Commitment average rating of (4). Faith average rating of (4). Kinship System average rating of (3.9).

The agreement made by the Delphi panel indicates that *belief system* and *faith* contributes to individual behaviors. Many Arab societies did not take preventive measures seriously because their members believed that the disease is the wrath of God that will only affect “infidel” societies or those that had been dominated by vice, injustice, and deviation from God’s rule. This belief continued even after the pandemic had spread throughout the world, including most of the Middle-East countries. Again, the belief of what God decreed is acceptable led to most people failing to take precautionary measures.

The panelists showed that individual economic status in general and *income status*, in particular, and effort to meet the living expenses of the family, remains important preventive behavior indicator. A large group of people and many segments with limited income associated with marginal and work professions fail to focus on social distancing and quarantine. The economic scenario was viewed as the indicator, but only for the poor and with low income. Parry et al. (2021) emphasized that poverty impacts the capabilities to provide medical protection. For instance, the economic scenario influences the possibility to provide major and continuous amounts of household and personal disinfectants and distribute face masks to individuals in the family more often (Platt et al., 2021). Alharbi (2021) added that in Arab countries, the national income level depends on individual countries. In turn, it demonstrated the ability of individual families to provide the needed resources for preventive steps in the form of masks, sanitizers, medical supplies, or health insurance. It also includes providing social security for compensation whenever there are cases of people needing financial support or losing their jobs. Many people in the Arab world are unable to meet their basic needs, making abiding by the COVID-19 preventive measure their last priority and concern.

One panelist stated, “There is no doubt that the *family commitment* in Arab countries surrounding individuals is deep and strong”. Not only because family is the first group in which the individual is raised, supported, and protected. But also, because, from a religious point of view, the parents are privileged in *Islamic Shariah* and the connection with them must be strong with no staleness. Numerous studies in the recent past have emphasized the importance of family as an important part of behavior prevention (Velleman et al., 2005; Sukar, 2020). This could have also resulted in several families disregarding social distancing and banning from social events in particular the bond between the elderly and children of the family, hence age in an indirect way has contributed to the prediction of protective behaviors. As a result of this, family relationships are natural, including the level of strength, intimacy, and stability directly impact the preventive behavior of individuals.

Regarding the *kinship system*, the argument is that the patriarchal persistence in Arab countries may present challenges for embracing protective behaviors, especially among women in rural areas as these women have limited access to information on the Internet on health awareness. Joseph (1996) emphasized that kinship values and relationships based on gender are crucial economically, socially, ideologically, politically, and mentally. The World Health Organization (2020) failed to consider the cultural variation when it came to formulating social distancing guidelines. As a result, it led to social abandonment of the guidelines, especially among communal families. Thus, the WHO was forced to make changes terming it “physical distancing” as opposed to “social distancing”.

### Psychological indicators

Findings from the Delphi panel identified four psychological indicators, namely Self-Efficacy with an average rating of (5). Perception of Hazard with an average rating of (4.5). Motivation with an average rating of (4.5). Stigma with an average rating of (4.2) (Table 3).

**Table 3:** The psychological indicators of individual Covid-19 preventive behavior.

Psychological indicators	Items	Average rating	Median rating	Average deviation	Minimum	Maximum
	Emotions: elevated anxiety will lead to rejecting the preventive measures	3.5	3	0.27	3	5
	Dramatic relief: having (friends or family members) infected with the COVID 19	2.5	2	0.45	1	3
	Health complication: prevention efforts are easier than any health complications from the virus	2.5	2	0.36	1	3
	Perception of hazard: elderly and at-risk people are more committed to preventive measures	4.5	4	0.09	2	5
	Self-efficacy: the personal perception that one is able to commit to all preventive measures	5.0	5	0.0	3	5
	Decision-control: individual control over decisions in commitment to the prevention of the emerging coronavirus	2.5	3	0.45	1	3
	Perceived cost: perception that following the prevention instructions is easy	3.0	3	0.0	3	5
	Positive attitude: perception that preventive behaviors are worth the effort	3.1	3	0.01	3	5
	Self-discipline: abide by the laws and regulations that protect against the Coronavirus	3.2	3	0.27	2	5
	Motivation: good quality of life motivates people to adhere to the guidelines to prevent coronavirus	4.5	4	0.09	3	5
	Motivation: following preventive guidelines is part of my family’s responsibility	3.2	3	0.55	2	5
	Stigma: fear of infection stigma motivates to adhere to prevention guidelines	4.2	4	0.18	3	5

Panelists identified the highest psychological indicator of *self-efficacy*, i.e., the perceived confidence of an individual to perform or depict a certain behavior as an important indicator of preventative behaviors. Self-efficacy has been explored in past research depicting several tendencies and health behaviors towards compliant attitudes and behaviors. Individual differences in cognitive abilities and self-efficacy potentially account for a certain level of difference in complying. Recent research supports this view as it relates to COVID-19 guidelines and recommendations, which include hygiene and social distancing. Individuals with a high level of perceived self-efficacy tend to be more associated with taking cautions, for example, sanitization, hand-washing, and social distancing (Blagov, 2021). It is unclear how this kind of behavior informs the compliance of the protective measures.

Kleitman et al. (2021) determined that personality openness was associated with taking a lot of precautions such as avoiding touch, washing hands, and social distancing. Compliance with recommendations requires people to prioritize rewards in the future such as flattening the curve over immediate and current rewards of non-compliance. This finding indicates that self-efficacy is in line with the idea that the perception of one capability to prioritize benefits over implications or costs may play a role in compliance with protective behaviors

Furthermore, the panelist identified psychological indicators related to the *perception of hazards* to individual and community health. The more people are threatened with the viral infection, the higher the commitment to adopt preventative behaviors is. The findings align with the Health Belief Model—the theoretical model identifies various components that facilitate individual change in behavior. These components include the perceived severity of the threat, perceived susceptibility to infection, perceived effectiveness of protective measures, and perceived self-efficacy in acting. The higher the perceived susceptibility to and severity of the threat and the perceived benefits and barriers of protective actions, the more likely health behaviors will be adopted (Okuhara et al., 2020). Threat perception encompasses beliefs about vulnerability to danger or harm from a disease and is associated with a variety of health behaviors. A notable component of risk perception is the level of fear or anxiety associated with the threat of a disease, as this is a strong motivator for influencing or changing behaviors. Studies show the relationship between worry about the disease and behavioral intentions developed by the theory of planned behavior. This is the case when it comes to COVID-19 as it relates to social distancing guidelines. (Ekta and Stephen, 2021).

*Motivation* is another psychological indicator indicated as a strong predictor of preventive behavior. Cook and Artino (2016) identified two forms of internal and external motivation. In this case, Arab communities constitute a major external motivation source where the person is forced to focus on preventive behaviors. In comparison, internal motivation originates from within the person and is based on an inner desire for satisfaction. The panelists’ report agreed on the essence of personal motivation, including the regulations for prevention, which form a primary driving factor towards the responsibilities of individuals. Further, motivation is related to feelings by which the individual is aware of the risks, feels fear, and is motivated to avoid harm by adhering to preventive behavior. Theories of health behavior indicate that the perspective of health threats constitutes a great motivator among the most fragile societal segments such as the elderly and those with chronic diseases, as they are expected to comply with social distancing, sterilization, and wearing a facemask more than others (Gehlert and Ward, 2011).

The notion of social *stigma*, as explored by the panelists, is double-edged. This is the case given that the stigmatization fear has prevented many and, thus, compel them to take control and preventative measures to avoid social distancing or isolation by other people, more so by their social environment and family (Norton et al., 2012). Furthermore, for the coronavirus, the discrimination associated with the disease, including the perceived risk, could be particularly relevant. At the same time, discrimination is a stigma feature and represents uneven treatment at both structural and individual levels, but with the objective to maintain privileges for individuals in a group (see Table 3).

The coronavirus pandemic has led to discrimination and social stigma against people based on perceived disease exposure and ethnic identities. An extensive body of research on the connection between health behaviors and stigma has been available. For instance, research on infectious conditions such as tuberculosis and HIV demonstrates that social stigma is related to disease testing, medication adherence, lower use of assistance, and disease disclosure (Kleitman et al., 2021).

## Discussion

Identifying COVID-19 preventive behavior indicators is a critical preliminary step in the development of programs for interventions related to pandemics. The scoping review identified a total of 24 preventive behavior indicators. The Delphi method and evaluation of the importance of these indicators by the panelists led to a consensus on 9 indicators, with the *belief system and self-efficacy* indicators receiving the highest average rating of 5 and the other indicators ranging between 3.9 and 4.5. The research aimed to define general sets of preventive behavior indicators, studies in the future could also apply these indicators in comparison to other various community-level behavior aspects during pandemics. Of importance is that the study findings are instructive to target public health interventions, which in turn, protect the lives of people in Arab countries. It included developing responses to the pandemic that is equitable, inclusive, and universal.

Most of the knowledge we have about individuals’ health behaviors during pandemics does not include accurate knowledge about Arab societies, although there are many studies that look at larger societies such as China, the UK, and the US (Torensma et al., 2021; Block et al., 2020). Therefore, this study will provide valuable insights into psychosocial factors that shape prevention behaviors in Arab countries to predict health behaviors in anticipation of future pandemics. Previous research on H1N1 indicated that human protective behavior during pandemics could be categorized into avoidance behaviors, preventive behaviors, and condition management (Kleitman et al., 2021).

The current research has identified several psychosocial predictive indicators related to COVID-19 preventive behaviors, these indicators could assist in developing prevention guidelines more suitable for Arab countries. Among the identified social indicators were those related to family ties, faith, and belief systems. Fincher and Thornhill (2012) indicates that throughout the world people differ in the magnitude with which they value strong family ties or heightened religiosity and how this affects adherence and stress management. Arab family ties are considered important in influencing individual behavior and adherence and these ties were strengthened during the COVID-19 lockdown. A study in Jordan showed that during the lockdown period, families were able to communicate and support each other and they mobilized faith and strong religious beliefs, to accept the pandemic crisis (Naser et al., 2021). This ethos needs to be integrated into pandemic intervention in Arab countries by developing health guidelines that to addresses the needs of families as a unit, not the individualistic approach. Mobilizing social modeling approach and religious values of kindes to one ‘s parent (*Bur Elwalden* in Arabic). Health education programs could address how families adhere to wearing masks and social distancing and how this preventive action is a family duty to protect its elderly.

Furthermore, social stigma was a strong barrier to help-seeking behavior and health adherence (Norton et al., 2012), health guidelines in Arab countries need to mobilize the deeply rooted faith values that prohibit discrimination and shunning of the ill and weak.

Economic status and income were also important indicators that need to be considered when imposing health recommendations in particular in Arab low-income countries. Where low-income individuals are not able to provide medication and food or inability to isolate themselves from members of the family, especially within the same household Abdelhafiz et al. (2020) indicates that while health guidelines have focused on the self-isolation process and imposed strict curfew. The need was to develop a sense of collectiveness facilitating altruistic behaviors like offering support and donations for daily-based workers.

Lastly, the current study indicated a unique combination of health behaviors predictors among Arab communities that included both self-efficacy and belief systems. These two micro-macro levels of human decision to comply or not comply (Kleitman et al., 2021) need to be considered when designing future prevention guidelines. By introducing a Community-Efficacy notion that emphasizes the collective responsibility to prevent future pandemics.

### Implications for policy and research

This study has certain implications for public health and health policy. The most important is that governments should increase public confidence in health guidelines and that future pandemic preventive measures need to adopt culturally sensitive approaches. Media-based awareness programs should tailor their messages to address social stigma, strengthen self-efficacy and motivation, and challenge community myths.

This study showed the potential to further investigate the impact of psychosocial characteristics on the adoption of prevention measures and risk perceptions by populations, as well as the role of context in Arab countries. Nonetheless, it was critical to understand more about the population, and individuals of lower psychological status, and how they have a lower perception to risk and take preventive measures. By understanding barriers and perceptions, interventions can be established for these groups backed up by evidence. Generally understanding how people perceive disease risk, and reacting to individual and system-level measures for future control of outbreaks is necessary, and can be acquired via qualitative research (Al-Hanawi et al., 2020).

### Strengths and limitations

One of the primary strengths of the study has been using an online data collection method that allowed it to overcome the challenges often experienced during in-person, one-on-one interviews, as well as limitations of mobility. The study also used a type of survey instrument, which applied to different disciples, while being accompanied by their views that allowed completion of the analysis, as well as increased the results’ credibility. The Delphi technique is limited in terms of its nature as it depends on the expertize of the panelists, which results in the instability of most members of the panel. The current research attempted to address this aspect through the identification of an interdisciplinary team to minimize instability and variation. This also included the determination of various psychological factors that follow the exclusion process. The current study did not consider demographic profiles such as gender and age, or education as a critical indicator for preventive individual behaviors that should be incorporated in future research should also incorporate age and gender in perceptions of COVID-19, especially around facilitators and barriers to adhering to the preventive measures.

## Conclusions

Determining indicators of preventive behaviors in the Arab community during COVID-19 is a necessary precursor to developing comprehensive intervention programs. A total of 9 indicators were explored as a representation of different perspectives regarding preventive behaviors. It was also determined how an expert panel had to use the Delphi method to review the list of different indicators before rating the importance of all the indicators in the assessment of preventative behaviors. The research explored a series of general preventive indicators and behaviors among the Arab community during the pandemic. Since consensus could not be reached for all indicators, further research is needed to determine how these indicators can be applied to different pandemics. Nonetheless, a comprehensive view and theory of how the preventive behaviors of communities should include more multi-layered indicators. The study provided a basis to advance the research on preventive behaviors even further, and identify and achieve consensus on critical indicators of the pandemic, as well as the associated behaviors.

## Data Availability

The datasets generated during and/or analyzed during the current study are available from the corresponding author upon reasonable request.
